# Early Development of an Innovative Nanoparticle-Based Multimodal Tool for Targeted Drug Delivery: A Step-by-Step Approach

**DOI:** 10.3390/cells14090670

**Published:** 2025-05-03

**Authors:** Chiara Barattini, Angela Volpe, Daniele Gori, Daniele Lopez, Alfredo Ventola, Stefano Papa, Mariele Montanari, Barbara Canonico

**Affiliations:** 1 Department of Biomolecular Sciences (DISB), University of Urbino Carlo Bo, 61029 Urbino, Italy; c.barattini@campus.uniurb.it (C.B.); daniele.lopez@uniurb.it (D.L.); stefano.papa@uniurb.it (S.P.); mariele.montanari@uniurb.it (M.M.); 2 AcZon srl, 40050 Bologna, Italy; angelavolpe@aczonpharma.com (A.V.); danielegori@aczonpharma.com (D.G.); alfredoventola@aczonpharma.com (A.V.); 3 Department of Pure and Applied Sciences (DiSPeA), University of Urbino Carlo Bo, 61029 Urbino, Italy

**Keywords:** ADC, nanomedicine, PSMA, MMAE, fluorescence, bioconjugation, nanoparticle, cancer, prostate

## Abstract

Prostate cancer is the most common tumor in men in developed countries and it often responds poorly to conventional treatments. Monoclonal antibody (MoAb) therapy, for this pathology, has grown tremendously in the past decades, exploiting naked and conjugated antibodies to cytotoxic payloads to form antibody drug conjugates (ADCs). Several studies have been carried out conjugating biomolecules against prostate-specific membrane antigen (PSMA), highly expressed in this tumor, to cytotoxic drugs. Nano-based formulations show high potential in targeted drug delivery to enhance the bioavailability of drugs. Our research aimed to evaluate the feasibility of setting up a nanoparticle-based multimodal tool for targeted drug delivery, describing the step-by-step approach and to perform a first screening of these fluorescent PEGylated silica nanoparticles employed in selective cancer cell targeting and killing. These nanoparticles featured a core–shell structure to contemporarily conjugate the antibody and the cytotoxic payload monomethyl auristatin E (MMAE) using a step-by-step approach. We compared the cytotoxic effect of this multimodal nanotool near the antibody-MMAE and free MMAE. We found a lower cytotoxicity effect of the nanoparticle-based construct compared to free drugs, likely because of the preservation of the previously observed receptor-mediated endocytosis. Nanomedicine is confirmed as a powerful alternative to organic drug delivery systems, even if some aspects, such as drug loading efficacy, release, scalable manufacturing and long-term stability, need to be deepened.

## 1. Introduction

Chemotherapy is one of the most important treatments for cancer diseases. However, the non-specific interaction of this class of drugs with healthy tissues, causing important side effects, represents one of the major shortcomings of this therapeutic approach. The targeted drug delivery of chemotherapy to tumors has been one of the major focuses of biomedical research since the early 20th century. Modern antibody drug conjugates are developed as “Trojan horses” to target a tumor-specific superficial antigen and to be internalized and metabolized in lysosomes releasing the payloads (more frequently ranging from one to eight drug molecules) which can reach their target district and elicit their specific function [1].

The last few decades have witnessed an intensive spread of nanomaterials which are increasingly becoming part of consumer goods, electronics, and biomedical tools due to their unique properties and applicability [2]. Nanoparticles (NPs) acquire different physicochemical properties in biological fluids, such as cell culture medium and blood. The surface of nanoparticles might be modified by the spontaneous coating of biomolecules, particularly proteins, which form the so-called “protein corona” [3]. As the cellular uptake of NPs strongly depends on their physical properties (e.g., size and shape) and chemical properties (e.g., surface charge), it is highly important to determine NP behavior in absence of targeting proteins on the shell [4,5]. It is particularly important in nanomedicine to improve the bioavailability of drugs [6]. Due to the enhanced permeability and retention (EPR) effect, the concentration of the nanoform-conjugated drugs in tumor sites increases because of the incompleteness of the newly formed tumor vessels [7].

Antibody–drug conjugated nanoparticles (ADCNPs) represent a new approach based on promising results achieved regarding both ADCs and nanotechnology, where the targeting specificity of antibodies are combined with the effector functionality of the nanoparticles. Conceptually, ADCNPs are similar to ADCs but, thanks to the presence of the nanostructure, an additional concentration effect can be exploited. The antibody conjugated on the NP recognizes its specific receptor expressed on the target cells, delivering the therapeutic agent in a controlled way and promoting its accumulation in the site where it is required while limiting off-target toxicity [8]. In the development of ADCNPs, we need to take into account challenges from new therapeutics, but also those related to the new manufacturing process, such as conjugation protocol optimization and quality control on final conjugates. All the manipulations, including protein-reduction, conjugation, and purification, could influence the activities of antibodies and drugs. To limit the impairments on antibody-conjugated nano-formulation, time-consuming development activities are needed, from conjugation condition optimization to purification protocol applications [9].

Nanomedicine has been extensively applied to monomethyl auristatin E (MMAE), a strong antimitotic agent which inhibits cell division by blocking the polymerization of tubulin and cannot be used as a drug itself because of its high toxicity. Cho and colleagues developed tumor-specific MMAE nanoparticles synthetized through a one-step synthesis exploiting the cathepsin B-specific cleavable and self-assembling FRRG (Phe-Arg-Arg-Gly) peptide [10]. Xie and co-workers synthesized PLGA-b-PEG _2000_ nanoparticles and loaded them with MMAE to improve the biosafety of MMAE and inhibit tumor growth [11]. Both these approaches exploited the enhanced permeability and retention (EPR) effect well-characterized in tumors.

**Figure 1:**
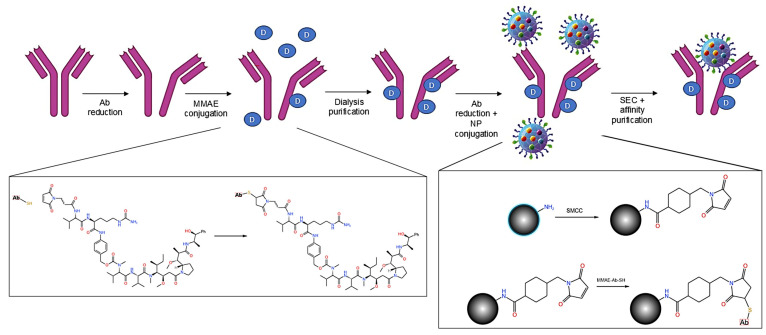
ADCNPs preparation; scheme of step-by-step approach adopted.

In this work, we developed a nanoparticle-based tool with contemporary fluorescent silica nanoparticles decorated with antibodies against prostate-specific membrane antigen (PSMA), highly expressed in prostate cancer cells and MMAE. For the antibody drug conjugation, we selected the valine–citrulline linker to allow the selective release of the drug in highly-cathepsin-B-expressing cells. The thiol–maleimide reaction was used to conjugate the ADC on the nanoparticles (Figure 1). In addition to the advantages due to nanoformulation, the exploitation of the intrinsic fluorescence of the nanoparticles lays the groundwork for taking advantage of optical imaging in drug delivery preclinical studies.

Indeed, we performed a first evaluation of the differences in cytotoxic effects among free MMAE, MMAE conjugated to anti-PSMA antibody, and MMAE conjugated to antibodies and nanoparticles on both PSMA positive and negative cell lines (respectively, LNCaP and MCF7). Such biological impact evaluations are intended as a first line of analysis and determine how to continue and modify the product and the study: further experiments are needed to obtain data on drug loading efficiency and release profiles that are able to deepen mechanistic explanations and critical for assessing the efficacy of the proposed system. Nevertheless, the present study is focused on the step-by-step approach in building the described nanoparticle-based multimodal tool for targeted drug delivery.

## 2. Materials and Methods

### 2.1. Fluorescent Silica Nanoparticle Synthesis

The nanoparticles employed in these studies were AcZon core–shell fluorescent silica nanoparticles. Nanoparticles NT_B_520 (λ excitation max 498 nm; λ emission max 520 nm) were synthesized according to the procedure previously described by Pellegrino et co-workers [12]. In brief, silica nanoparticles were synthesized through a one-pot, two-step reaction described as a micelle-assisted method [13,14]. All reagents (including fluorescent dyes) are mixed in a solution of water and n-butanol. In this condition, the surfactant (Brij-58, Merck Millipore, Burlington, MA, USA) creates micelles within which all reagents (previously modified by the addition of trialkoxysilane groups), because of their hydrophobicity, are spontaneously arranged.

When ammonia, the catalyst, and the silane precursor are added to the mixture, the base-catalyzed hydrolysis of all trialkoxysilane groups takes place, forming core–shell fluorescent silica nanoparticles. The shell is composed of two different polyethylene glycols terminating with a trialkoxysilane group, one inducing stability and solubility in an aqueous environment, while the other is functionalized with amine reactive groups allowing for subsequent biomolecule conjugations.

### 2.2. Monoclonal Antibody Production

The hybridoma CB-PSMA derives from cells from an immunized BALB/c mouse, fused to NS0 mouse myeloma cells, according to Köhler and Milstein technique and stored in a nitrogen liquid bank [15]. Cells were thawed in a 12-well plate in RPMI-1640 with stable L-glutamine (Euroclone, Pero, Italy) supplemented with 10% heat-inactivated fetal bovine serum (FBS) (Sigma-Aldrich, St. Louis, MO, USA) and 1% penicillin-streptomycin (Sigma-Aldrich, St. Louis, MO, USA). Once in the logarithmic growth phase, cells were transferred to T-flasks with a lower percentage of FBS (5%) up to viable cell total number was equal to 2 × 10^8^ cells, counted using Trypan blue exclusion assay (Thermo Fisher, Waltham, MA, USA) on a Neubauer chamber. Cells were then centrifuged, and the pellet was resuspended and seeded in a special EZ-flask (KDBio, Berstett, France), featured by a gas permeable membrane at the bottom of the flask, in 1 L of complete medium. Hybridomas were left in the incubator in standard conditions (37 °C under a humidified controlled atmosphere with 5% CO_2_), and the supernatant, containing monoclonal antibodies, was harvested after 30 days from the inoculum (Figure S1).

The supernatant was centrifuged at 700× *g* 5 min at room temperature, then filtered with 0.22 µm polyether sulfone (PES) filters (Nalgene, Rochester, NY, USA). The filtered supernatant was applied on the MabSelect™ SuRe column (Cytiva, Marlbourough, MA, USA) using a bidirectional pump for low-pressure protein purification (Bio-Rad, Hercules, CA, USA). The monoclonal antibodies were eluted from the column using citrate buffer (0.1 M—pH 3.0) and rapidly buffered with a 15% volume of Tris-HCl (1 M—pH 9.0). The obtained mixture was dialyzed against phosphate buffer (10 mM NaPi/150 mM NaCl pH 7.35 (±0.05)) under stirring at 2/8 °C for at least 48 h with a ratio sample: buffer higher than 1:20 [16].

### 2.3. Antibody to Nanoparticle Conjugation

Briefly, the conjugation of the mouse anti-human PSMA to fluorescent silica nanoparticles exploits the reduction of antibody interchain disulfide bonds to form reactive cysteine residues for coupling with maleimide groups to generate a thiosuccinimide product, as shown in Figure S2 [17].

Nanoparticles were activated by their prearranged conjugation to an 8.3 Å heterobifunctional crosslinker, named succinimidyl-4-(N-maleimidomethyl)cyclohexane-1-carboxylate (SMCC, Setareh Biotech, Tyler, TX, USA), featured by a N-hydroxysuccinimide (NHS) ester and a maleimide group, to react with cysteines on reduced antibodies and, on the other end, to NH_2_ groups on NPs. A mass of 32 mg of NT_B_520 (corresponding to 5 mL), previously transferred by ultrafiltration in 50 mM NaPi/1 mM EDTA (ethylenediaminetetraacetic acid) pH = 8, was made to react with 10 mM of SMCC, dissolved in dimethyl sulfoxide (DMSO), for 60 min at room temperature. The mixture was then purified by SEC using 50 mM 2-(N-morpholino)ethanesulfonic acid (MES)/2 mM EDTA pH 6.0, to remove the unconjugated crosslinker.

On the other hand, the disulfide bonds in the hinge region of the antibody were cleaved using a mild concentration (20 mM) of 1,4-dithiothreitol (DTT, Sigma-Aldrich, St. Louis, MO, USA) for 60 min, under stirring, in the dark, to control the reaction. A desalting on the Sephadex G25 medium column was used to separate reduced and non-reduced antibodies.

**Figure 2:**
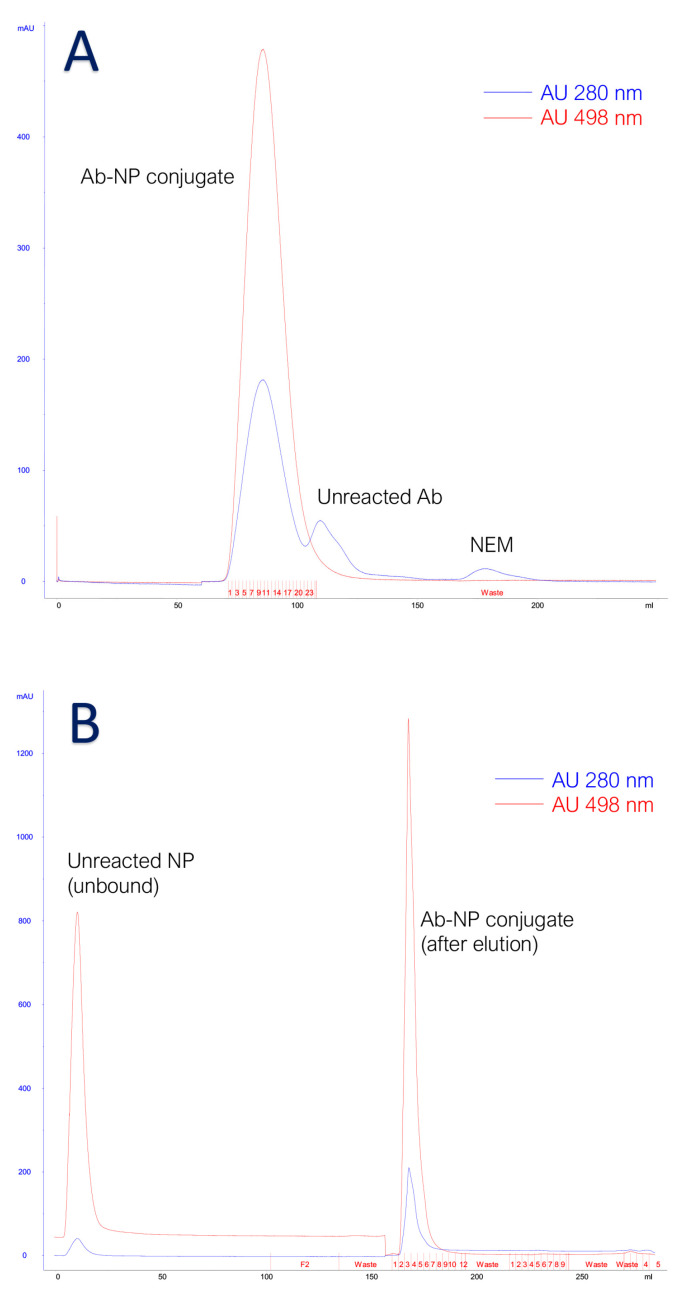
(**A**) SEC chromatogram. Blue line represents absorption at 280 nm and red line at 498 nm. (**B**) Protein G chromatogram. Blue line represents absorption at 280 nm and red line at 498 nm.

The reduced antibody and the activated nanoparticles were incubated, with a molar ratio NP:MoAb equal to 2, for 60 min at room temperature, in the dark, under gentle agitation. This molar ratio resulted in the best one to obtain an average of 1 nanoparticle conjugated to each antibody and to reduce the number of unreacted antibodies in the mixture. An excess of N-ethylmaleimide (NEM, Sigma-Aldrich, St. Louis, MO, USA) was added under agitation to stop the conjugation reaction. NEM forms stable, covalent, thioether bonds with sulfhydryl groups (e.g., reduced cysteines), enabling them to be permanently blocked to prevent disulfide bond formation. A two-step purification protocol was adopted to remove the free species, unreacted antibodies and nanoparticles, from the conjugated pool. The mixture was firstly purified by size exclusion chromatography on Superdex™ 200 high resolution gel filtration for molecular weights between 10 and 600 kDa (globular proteins) (Cytiva, Marlbourough, MA, USA), packed on a XK16 column (Cytiva, Marlbourough, MA, USA) mounted on AKTA purifier 10 HPLC System (Amersham, Buckinghamshire, UK). This first step allowed for the separation of free and conjugated nanoparticles from free antibodies due to the significant difference in mass. Conjugated and free nanoparticles, bigger than free antibodies, were less retained by the resin and were eluted in a shorter time (Figure 2A). Unreacted NEM (molecular weight 125.13 g/mol) is the smallest mixture species and eluted last. Then, the resulting pre-purified mixture was applied on an affinity chromatography column packed using Protein G Sepharose Fast Flow 4 (Cytiva, Marlbourough, MA, USA) as resin. The adopted stationary phase was a recombinant protein G lacking an albumin-binding region crosslinked to an agarose matrix. The protein G has a strong affinity for the Fc antibody fragment so only the species with Fc fragment were retained by the resin and eluted with low pH (Figure 2B). The conjugated antibodies were eluted from the column using a glycine solution (0.1 M, pH 2.40 ± 0.05) and immediately buffered with Tris-HCl (1 M, pH 9.5 ± 0.1) in a proportion equal to the 10% of each eluted fraction volume, then dialyzed against phosphate buffer using regenerated cellulose membrane with a cut-off of 50 kDa (VWR, Radnor, PA, USA) under gentle stirring for at least 24 h. The conjugate was then concentrated using Amicon stirred cell (Merck Millipore, Burlington, MA, USA) and PVDF cut-off 50 kDa membrane (VWR, Radnor, PA, USA) under nitrogen flux, and centrifuged at 3000× *g* 30 min at 4 °C to discard the eventual pellet.

### 2.4. Antibody Drug Conjugation

One of the most important features in ADC preparation is the rigorous control of the drug molecules bound for each antibody. If a low number of molecules might result in inefficient treatment, it is proved that an elevated drug-to-antibody ratio (DAR) could lead to ADC instability. The conjugation protocol applied is a site-directed cysteine-based conjugation historically used to obtain homogeneous ADC. To control the final DAR, we applied Ellman’s assay, developed in 1959, to strictly quantify -SH groups available on antibodies after their reduction [18].

**Figure 3:**
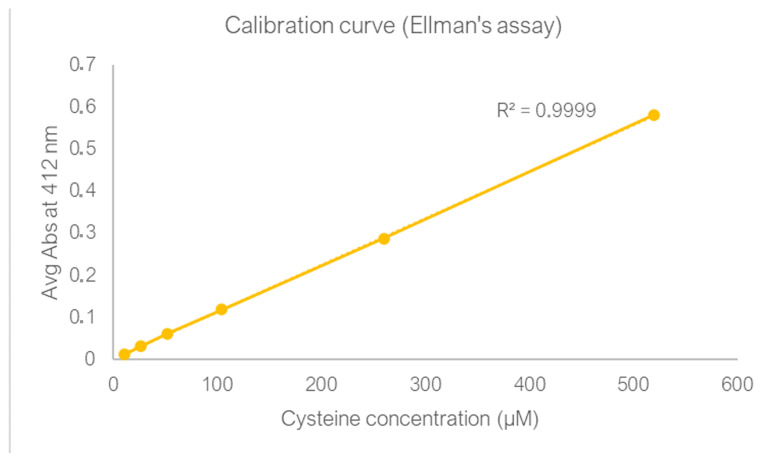
Linear standard curve to determine cysteine concentration.

The antibody mouse anti-human PSMA was transferred by ultrafiltration in the reduction buffer (NaPi/EDTA pH 8.0) at a final concentration of 2.5 mg/mL. A total of 3 equivalents of the reducing agent TCEP (tris(2-carboxyethyl)phosphine, Sigma Aldrich, St. Louis, MO, USA) were added at controlled temperature (37 °C in a thermostatic bath) as this condition was assessed as most favorable in previous experiments [19]. After 1 h of incubation, Ellman’s assay was performed to quantify the free sulfhydryl groups available for bioconjugation. A sample of the reduced antibody was incubated for 15 min at room temperature, in the dark, with 10 µL of a 4 mg/mL solution of Ellman’s reagent, also known as DTNB (5,5′-dithiobis(2-nitrobenzoic acid), Sigma Aldrich, St. Louis, MO, USA). The yellow-colored compound, 5-thio-2-nitrobenzoic acid (TNB), derived from the reaction of the free sulfhydryl groups with the Ellman’s reagent, is observed spectrophotometrically by measuring absorbance at 412 nm. Interpolating this value on a previously constructed calibration curve using known quantities of cysteine (Figure 3), we obtained an average of 5.3 free -SH groups on each antibody. To conjugate one molecule of the drug for each -SH group, we adopted the molar ratio of 6 for the conjugation of the antibody to MMAE.

We selected a ready to conjugate form of the drug: VcMMAE (mc-vc-PAB-MMAE, MedChemExpress, South Brunswick, NJ, USA) in which MMAE is linked via the lysosomal cleavable dipeptide valine–citrulline (vc) to the maleimidocaproyl (mc), which is one of the most used maleimide-type linkers, exhibiting an excellent reactivity with sulfhydryl groups.

The conjugation reaction took place at room temperature for 60 min under rotating stirring. At the end of the incubation period, the reaction was stopped by the addition of an excess, 20 equivalents calculated against MMAE, of cysteine (Sigma Aldrich, St. Louis, MO, USA).

To eliminate the unreacted drug and the free cysteine, the conjugated antibodies were dialyzed against phosphate buffer for 24 h at 2/8 °C using a regenerated nitrocellulose membrane with a cut-off of 50 kDa (Spectrum Labs, San Francisco, CA, USA). After the recovery from dialysis, Ellman’s assay was repeated to assess the presence of free sulfhydryl groups which were not significantly found.

### 2.5. Antibody Drug Nanoparticle Conjugation

**Figure 4:**
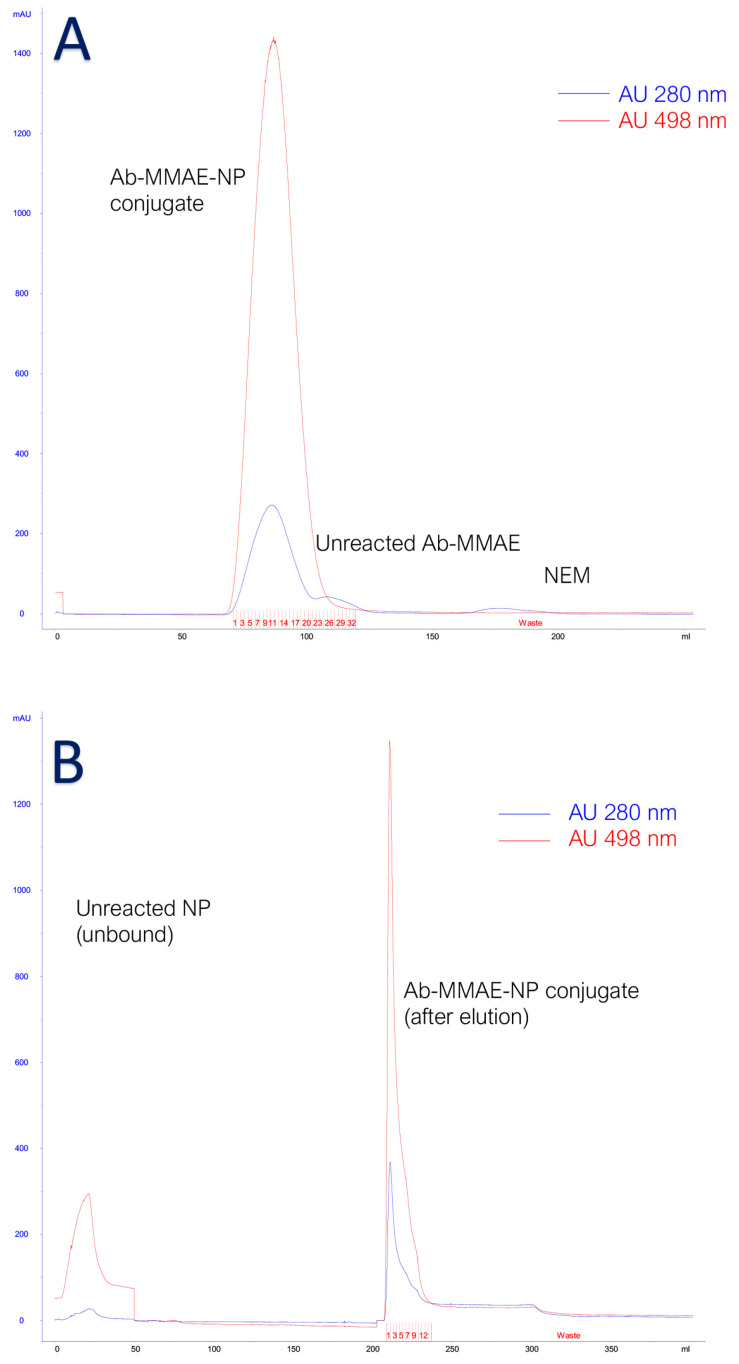
(**A**) SEC chromatogram. Blue line represents absorption at 280 nm and red line at 498 nm. (**B**) Protein G. Blue line represents absorption at 280 nm and red line at 498 nm.

We combined the conjugation procedures previously developed in this study to obtain the multimodal tool for NP-antibody and MMAE-antibody conjugations. Briefly, the mouse anti-human PSMA conjugated to MMAE was newly reduced with 3 equivalents of TCEP for 30′ with the aim of obtaining new -SH groups available for nanoparticle conjugation. We decided to employ TCEP instead of DTT, which is used in the NP-Ab conjugation, to avoid the purification step which would cause an additional decrease in the final yield. The availability of free reactive sulfhydryl groups was confirmed, as expected, by Ellman’s reaction. Then, the reactivated antibody was mixed with SMCC-activated nanoparticles in a molar ratio NP: Ab 2:1, for 60 min, in the dark under stirring. The reaction was stopped by adding an excess of NEM, and the obtained products were submitted to the multimodal chromatography optimized protocol (size exclusion chromatography and protein G affinity chromatography as in Figure 4) to ensure the highest purity possible.

### 2.6. Cell Culture

The experiments were carried out on two different cell lines with no expression and high expression of PSMA antigen: MCF7 (ATCC HTB-22™, LGC Standard, Middlesex, UK) negative for the antigen of interest and LNCap cell line (ATCC-CRL-1740™, LGC Standard, Middlesex, UK) which strongly expresses PSMA antigen [20,21].

MCF7 cells were cultivated using RPMI 1640 with stable L-glutamine (Euroclone, Italy) with 1% penicillin/streptomycin (Sigma Aldrich, St. Louis, MO, USA) and 10% heat-inactivated FBS (Sigma Aldrich, St. Louis, MO, USA). After thawing, cells were cultivated under standard conditions (37 °C under a humidified controlled atmosphere with 5% CO_2_) and sub-cultured when the confluence reached 80/90% and/or the color of the culture medium turned dark yellow.

LNCap cell line, clone FGC (fast growing clone, ATCC CRL-1740™, LGC Standard, Middlesex, UK) was cultured strictly following the supplier’s instructions. Cells were seeded in a boosted RPMI-1640 medium (Pan Biotech, Aidenbach, Germany) containing 2 mM L-Glutamine, 1 mM pyruvate, 4.5 g/L glucose, 10 mM HEPES, and 1.5 g/L NaHCO_3_. This medium was supplemented with 10% non-heat inactivated FBS and 1% penicillin/streptomycin. This cell line formed weakly adherent monolayers easily managed by tapping, shaking, or pipetting to detach. According to the literature and manufacturer instructions, the reattachment of these cells was slow, and about 70% of the initial cells adhered to the growth surface within 48 h.

The day before the treatment, both cell lines were detached using trypsin-EDTA 0.25% solution (Sigma Aldrich, St. Louis, MO, USA) and counted through the Neubauer chamber (Sigma Aldrich, St. Louis, MO, USA) under a light microscope Optiphot 2 (Nikon, Tokyo, Japan). According to the literature, MCF7 and LNCaP cell doubling times are, respectively, 24 h and 60 h [22,23]. To obtain approximately the same number of cells at the moment of the treatment, we decided to seed 100,000 MCF7 and 200,000 LNCaP separated in each of the 24 chambers of 4-well glass chamber slides in the smallest volume of complete medium. After a few hours, when the adhesion process was clearly begun, the final volume was adjusted to 100 µL. Cells were maintained overnight in standard conditions.

### 2.7. Cell Treatment

According to the literature, the IC_50_ of MMAE in the MCF7 cell line and LNCaP is 0.5 nM and 1.06 nM, respectively [24,25]. We decided to investigate three different drug concentrations: 0.5 nM, 5 nM, and 50 nM, with the aim of understanding if the presence of additional species (antibodies and antibodies plus nanoparticles) might have an impact on the half-maximal inhibitory concentration and/or in the time of action. It is important to highlight that, the dilutions were prepared using a complete cell culture medium at the lowest MMAE concentration examined. Two different negative controls were added to the experiment: complete medium, representing optimal cell culture conditions, and 0.5% dimethyl sulfoxide (DMSO vehicle) diluted in culture medium, representing the highest possible residual quantity of DMSO among the conjugates under investigation, although due to multiple dialysis step, the presence of traces of this substance was remote.

According to the literature, we considered three different incubation time points: 24, 48, and 72 h [10,26,27].

On the day of the treatment, cells were checked by inverted microscope, then the exhausted medium was aspirated, and cell layers were gently washed twice with sterile PBS (phosphate-buffered saline, 10 mM NaPi, 150 mM NaCl, pH 7.3–7.4 reagents purchased from Sigma Aldrich, St. Louis, MO, USA).

Then, each treatment was performed (previously diluted to a final volume of 100 µL). Treated cells were left in standard conditions for the abovementioned time course.

### 2.8. Viability Assay

Once the incubations were over, cell status was rapidly checked under the inverted microscope. Then, the treatments were removed and collected in a centrifuge tube. Cell layers were gently washed once with sterile PBS. Cells were detached using trypsin/EDTA. This cell suspension was washed with sterile PBS and, after centrifuge, resuspended in PBS. This step was fundamental to avoid possible bias due to dead detached cells in the exhausted medium (especially in those cases where the survival rate was poor). Cell viability was assessed using exclusion trypan blue viability assay (dilution 1:10 in trypan blue 0.4%, Sigma Aldrich, St. Louis, MO, USA). For each experimental point, three different samples were prepared to be independently submitted to trypan blue assay [28]. Cell viability was calculated using the formula:

$$Cell\;viability=\frac{total\;cells-dead\;cells}{total\;cells}*100$$

The survival rate was calculated using the formula:

$$Survival\;rate=\frac{treated\;cell\;viability}{untreated\;cell\;viability}*100$$

where “untreated cells” were represented by cells in complete, standard cell culture medium.

## 3. Results

### 3.1. Characterization of Fluorescent Silica Nanoparticles

**Figure 5:**
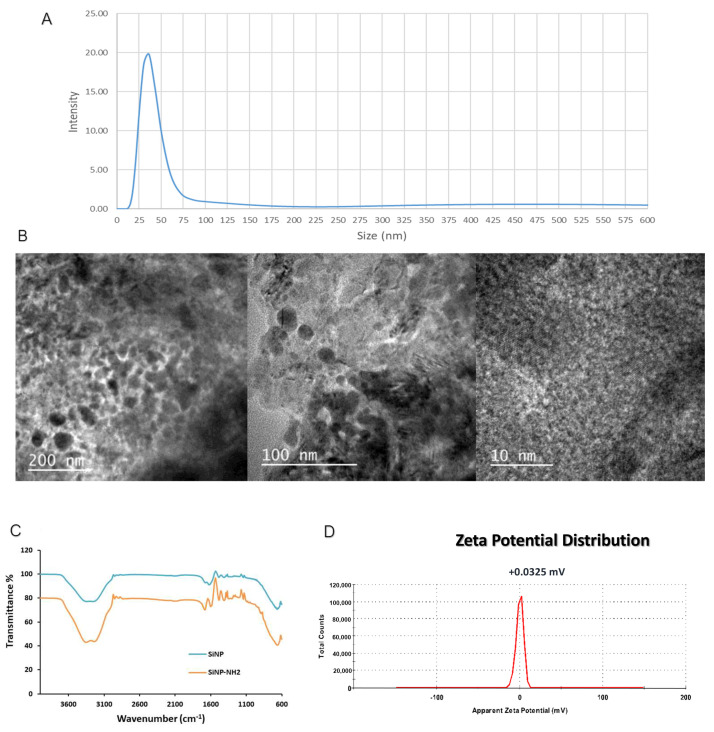
Characterization of NTB520 by (**A**) DLS; (**B**) transmission electron microscopy; (**C**) FTIR; (**D**) Z potential.

**Figure 6:**
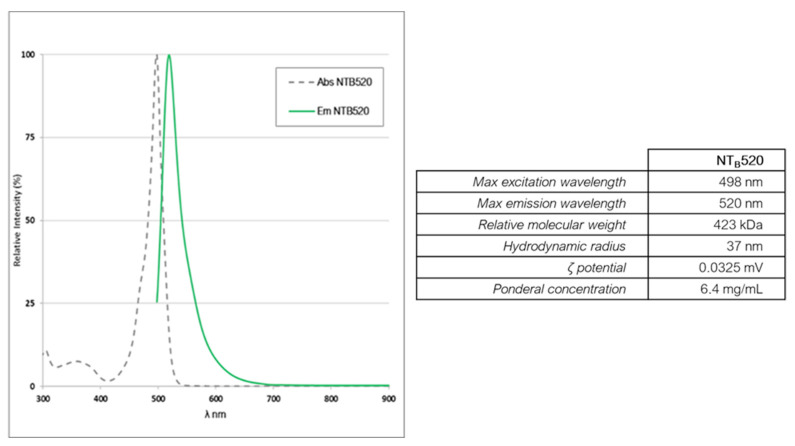
Absorption and emission spectra of NTB520 together with main photophysical features.

Nanoparticles were characterized for their ponderal concentration using vacuum centrifugation (Heidolph, Schwabach, Germany), the polydispersity index was evaluated using dynamic light scattering (DLS) (Malvern Panalytical, Grovewood Road, UK), the morphology was assessed by transmission electron microscopy (TEM, Jeol, Tokyo, Japan), and the surface was investigated with Fourier-transform infrared spectroscopy (FTIR) (Perkin Elmer, Waltham, MA, USA). Characterization results are reported in Figure 5 and Figure 6.

The images collected from the TEM analysis show a strong degree of aggregation in addition to something similar to a coating covering the whole sample, probably due to the presence of surfactant residues in NP mixture which form this layer once the samples were dried during its preparation for microscopic analysis [29].

The relative molecular weight was assessed using size exclusion chromatography dealing with nanoparticles as if they were globular proteins. The resin used was Superose 6 (Cytiva, Marlborough, MA, USA) [30]. The DLS analysis was employed to evaluate the hydrodynamic radius and ζ potential which was near to zero as this kind of nanoparticles are stable in water solution due to hydrogen bonds formed by PEG chains [31]. The FTIR spectrum, executed on functionalized and not functionalized nanoparticles, reflects the composition of the nanoparticles themselves [32].

The number of dyes (F) entrapped in each NT_B_520 nanoparticle (NP) resulted in a value of 15.8, estimated according to spectrophotometric measurements according to the formula:

$$\frac{F}{NP}=\frac{{µM}_{donor}}{{µM}_{NP}}$$

where

$${µM}_{donor}=A_{498}*\frac{1}{\varepsilon_{donor}}$$

and

$${µM}_{NP}=\frac{Ponderal\;concentration}{Relative\;molecular\;Weight}*1000$$

The quantification of the reactive amines available on nanoparticle surface was obtained using a colorimetric indirect method based on the stoichiometric ratios of the reaction between NH2 groups, available on NP shell, and the isothiocyanate of fluorescein isothiocyanate (FITC, Sigma Aldrich, St. Louis, MO, USA). This reaction took place with a molar ratio of 10 and after 1 h of incubation, and the unreacted FITC was removed by desalting the Sephadex G25 column (Cytiva, Marlborough, UK). The maximum absorption wavelength of FITC was directly proportional to the amine groups available on nanoparticles for further conjugations.

### 3.2. Characterization of Antibody Conjugated to Fluorescent Silica Nanoparticles

**Figure 7:**
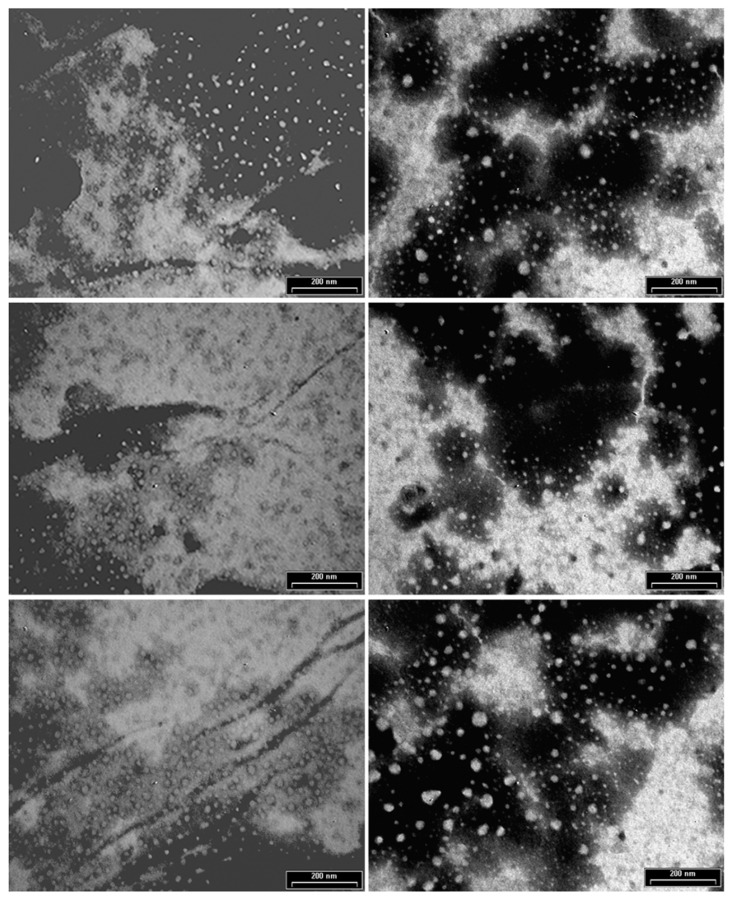
Transmission electron microscope analysis of stand-alone NPs (**left**) and SMCC-conjugated NPs (**right**).

Activated (conjugated to SMCC) and non-activated nanoparticles were investigated by means of transmission electron microscopy (Figure 7). The morphological analysis showed an enhancement of the aggregation status of the nanoparticles once pre-conjugated with SMCC, probably due to additional interactions caused by spacer arms. This aggregation is present, to a lesser extent, even in non-activated nanoparticles, which might be attributed to the sample preparation, especially to the dehydration needed for electron microscope analysis. No evidence of this aggregation was shown in dynamic light scattering analysis when nanoparticles were in their original condition, in aqueous buffer.

In accordance with spectrophotometric characterization, about one nanoparticle has been conjugated to each antibody (NP/Ab ratio 1:1) corresponding to dye/Ab ratio of 15.8:1. These data are obtained using the formulas below:

$$\frac{NP}{Ab}=\frac{{µM}_{NP}}{{µM}_{Ab}}$$

where

$${µM}_{NP}=\frac{A_{498}*\frac{1}{\varepsilon_{498}^{donor}}}{\frac{F}{NP}}$$

and A_498_ is the maximum absorption at the specific wavelength and epsilon (ε) is the molar extinction coefficient of the species at the specific wavelength.

### 3.3. Characterization of Antibody Conjugated to Monomethyl Auristatin E

**Figure 8:**
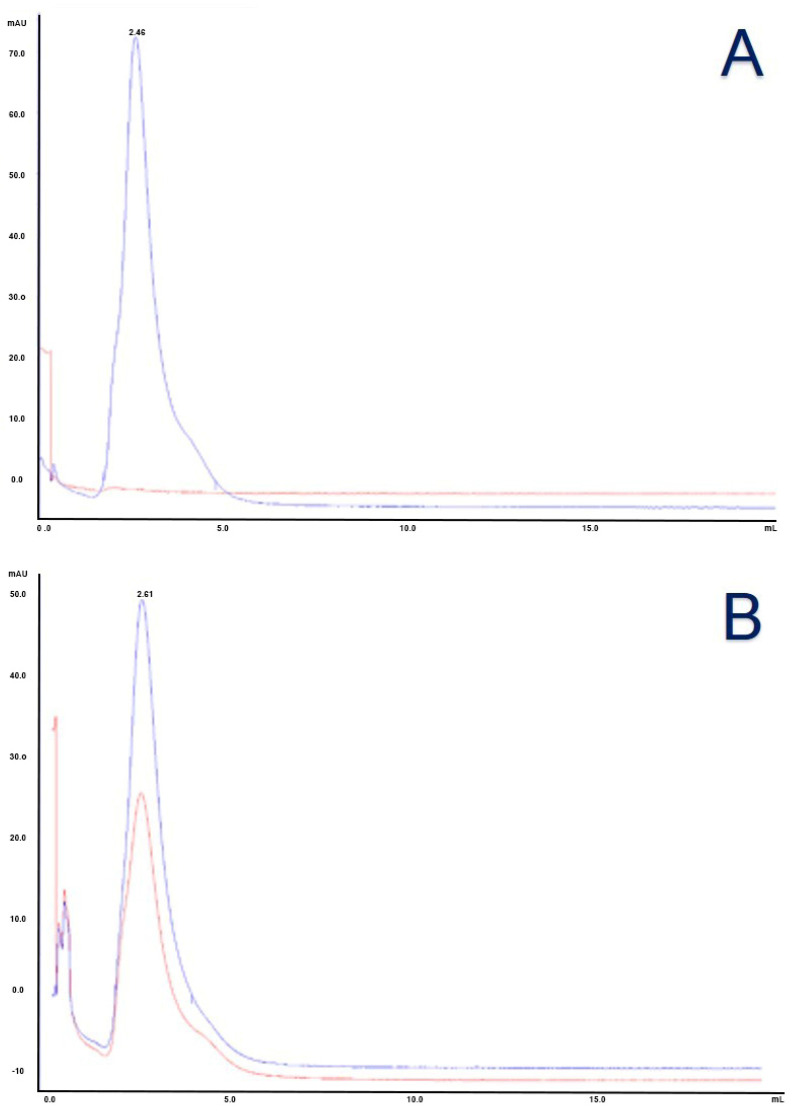
(**A**) SEC chromatogram of unconjugated anti-PSMA. Blue line represents absorption at 280 nm and red line at 248 nm. (**B**) SEC chromatogram of anti-PSMA-MMAE. Blue line represents absorption at 280 nm and red line at 248 nm.

A size exclusion chromatography was performed to evaluate the presence of free species, which were not detected as depictable from chromatograms in Figure 8. In accordance with the calibration kit ranging from 1.35 to 670 kDa (Bio-Rad, Hercules, CA, USA) employed to evaluate molecular weight with size, the conjugated antibody is featured by a molecular weight of 223 kDa higher than the unconjugated antibody, thus likely related to the conjugated form (elution volume of purified antibody 2.61 mL—calibration kit referral for 158 kDa gamma globulin is 2.56 mL, against 2.46 mL of the conjugated one) [33].

**Figure 9:**
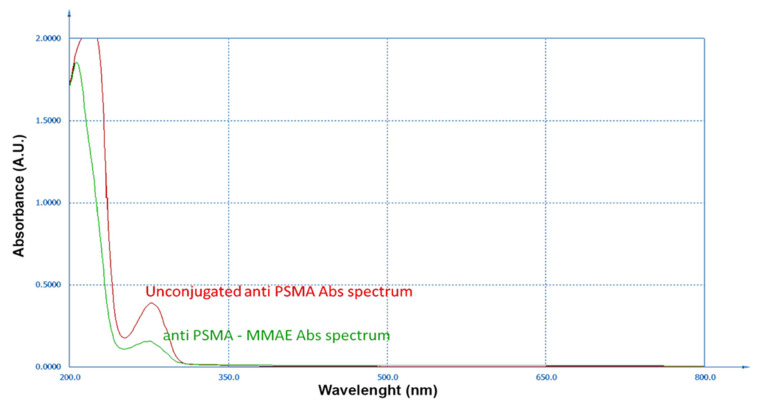
Overlayed absorption spectra of unconjugated anti-PSMA (red line) and anti-PSMA MMAE (green line).

The DAR quantification was particularly problematic due to the nearness of the maximum absorption of the antibody (280 nm) and MMAE (248 nm), which were not clearly separable due to the interference of the used buffer. Considering the contribution at 280 nm of exclusive pertinence of the antibody, from the absorption spectrum in Figure 9, and in accordance with the method described by Cruz and colleagues, considering the molar weights of antibody and MMAE (respectively 150 kDa and 1316.63 g/mol) and the related extinction molar coefficients (ε) at 280 nm (antibody 21,000 M^−1^cm^−1^ and MMAE 1500 M^−1^cm^−1^) and 248 nm (antibody 77,500 M^−1^cm^−1^ and MMAE 15,900 M^−1^cm^−1^), we calculated a A_280/248_ = 1.75, corresponding to a DAR equal to 2.8, consistent with DARs reported for similar ADC synthesis methods [34,35,36].

### 3.4. Characterization of Antibody Conjugated to Fluorescent Silica Nanoparticles and Monomethyl Auristatin E

We calculated the molar concentration of MMAE using the formula:

$${µM}_{MMAE}=\frac{\varepsilon_{248}^{Ab}*A_{280}-\varepsilon_{248}^{Ab}*A_{248}}{\varepsilon_{280}^{MMAE}*\varepsilon_{248}^{Ab}-\varepsilon_{248}^{MMAE}*\varepsilon_{280}^{Ab}}$$

where epsilon (ε_XXX_) is the molar extinction coefficient of each species (indicated uppercase) at the specific wavelength and A_XXX_ represents the maximum absorption at the specific wavelength. As stated by the reported formula the MMAE concentration in the conjugate was 1.32 µM.

### 3.5. Comparison of Different Conjugates Toxicity Effects on Cell Lines

The incubation of cells with the highest dose possible of DMSO did not cause cytotoxic effects with a survival rate not lower than 98% as foreseen. As a result, survival rates observed during the incubation are exclusively attributable to the effect of MMAE in all its forms.

Free MMAE is confirmed to have a strong cytotoxic effect and, according to the literature, MCF7 cell line resulted more sensitive to low doses and short incubation times, confirming that the cytotoxic effect of the free drug is unrelated to PSMA expression.

**Figure 10:**
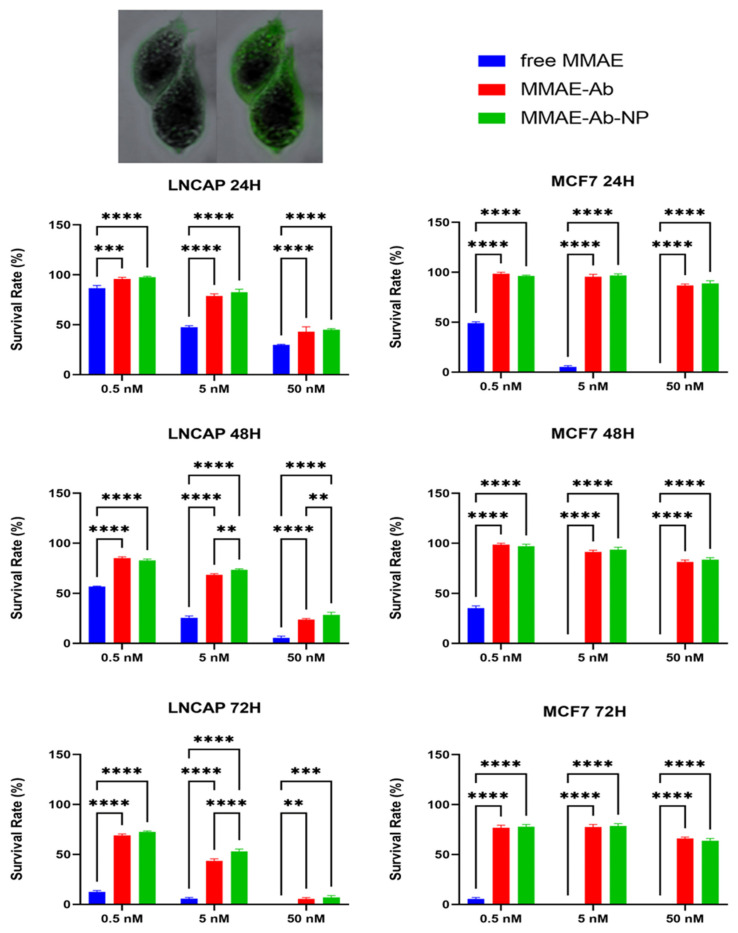
Statistical histogram of survival rate of LNCaP (on **left**) and MCF7 (on **right**). Two-way ANOVAs were followed by a Tukey post hoc test. In the box, 40× magnification of trypan blue staining (**left**) and fluorescence due to presence of fluorescent nanoparticle-based complex (**right**) A two-way analysis of variance (ANOVA) with a Bonferroni post-hoc comparison revealed a statistically significant result: ** = *p* < 0.01, *** = *p* < 0.001, **** = *p* < 0.0001.

The conjugation of the targeting molecule, anti-human PSMA, to the effective drug inhibits the cytotoxic effect in MCF7 which does not express the targeted antigen. A significant effect has been shown to be caused by both conjugates (with and without nanoparticles) in MCF7 at the highest dose at the longest incubation time. The fluorescence image confirmed the presence of the nanoparticle-based compound inside the cells while they do not express the target antigen. Even if the caused effect is not comparable to the one induced in PSMA-expressing cell, the difference in survival rate between 72 h of incubation and shorter periods is worth the attention. The effect of the conjugated drugs (in both formulations) in LNCaP followed the dose/effect model, as expected. Data are graphically reported in Figure 10.

## 4. Discussion

Our research aims to evaluate the feasibility of setting up a drug delivery system starting with functionalized silica nanoparticles, combined with both a drug and a specific antibody. This multimodal tool is constructed step by step (from Section 2 and Section 3), and a first screening was conducted about the possibility of employing fluorescent PEGylated silica nanoparticles in selective cancer cell targeting and killing. Our results demonstrated that no specific effect was attributable to DMSO (vehicle). On the other hand, free MMAE resulted as a potent cytotoxic drug without any selectivity, proving its non-applicability as a stand-alone drug, as extensively depicted in the literature and demonstrated by the lowest survival rates of both cell lines to the free drug. No appreciable differences in toxic effects were observed between the conventional ADC and the innovative ADCNPs. This behavior is probably due to the conjugation method, which did not enhance DAR as expected. Another approach is now under evaluation to increase the number of drug residues per complex. This method needs an additional crosslinker, a thiol PEG-NHS, to diversify reactive groups on nanoparticles. Through the application of this new moiety, nanoparticle amine groups will be contemporarily functionalized with maleimide groups to link reduced antibody -SH and with -SH groups to bind maleimide groups available at the end of the MMAE crosslinker. This method is still under development.

As expected, the conjugation of a targeting antibody to highly cytotoxic drugs confer specificity to the cytotoxic effect of the drug itself, confirming the multimodal tool’s effectiveness. The differences in the survival rates between expressing (LNCaP) and not expressing (MCF7) PSMA antigen are evident after 24 and 48 h of treatment with 5 nM and 50 nM treatments, and after 48 h even with the less concentrated one (0.5 nM).

Interestingly, at the longest time of incubation (72 h) and highest concentration (50 mM), the targeting efficacy of the tool seems to be reduced, as the death of non-expressing PSMA cells slightly increased with respect to shorter incubation times. Several hypotheses might be formulated to explain this peculiar feature. It is likely that, at a longer incubation period, cells basally reveal a higher degree of cell death, independent from the drug itself. Indeed, lysosomal enzymes, such as cathepsins, might be released following cell death, in the extracellular environment and cleave the sensitive linker, causing drug release [37]. Due to the unwanted release of lysosomal enzymes, this drug activation leads to an undesirable bystander-like effect, killing even MCF7 cells that do not express the antigen recognized by the antibody and moving to a condition similar to the one with free MMAE [38]. These speculations are intended as limitations of the current study; however, as previously cited, the main aim of this work was to describe the step-by-step approach to building a nanoparticle-based multimodal tool for targeted drug delivery. Although in vivo evaluations, with appropriate model selection, appear mandatory in this kind of study, it is noteworthy that tests on animals are always conducted after having gathered essential and straightforward data in more controlled in vitro systems. Nevertheless, the literature reports that valine–citrulline linkers are stable in cynomolgus monkey and human plasma, but not in mice; appropriate appraisals or linker modification need to be evaluated before proceeding [39,40].

The choice to use maleimide chemistry for the conjugations is due to the need for controlled and site-specific conjugation by reducing disulfide bonds, particularly in the Fc region in the antibody, to render thiol groups accessible [41]. Regrettably, one of the main drawbacks to consider is the ADC preparation via maleimide–thiol conjugation, namely the retro-Michael addition [42]. This reaction results in the exchange of the linker drug with thiol-containing molecules such as glutathione or albumin, causing the loss of targeting activity as described by Lu’s group in 2015 [43]. Lahnsteiner and colleagues suggest stabilizing the thiosuccinimide via a trans-cyclization to avoid this issue [44]. The group of Fontaine, instead, offers two alternative solutions: the employment of a succinimidyl–thioether conjugate with a high ratio of hydrolysis to exchange rate, and the use of a maleimide analogue to form a rapidly hydrolyzed succinimidyl–thioether to prepare conjugates, then hydrolyzing the succinimidyl–thioether in the absence of thiols before in vivo dispensation [45]. On the other hand, in recent years, several alternative conjugation methods to obtain stable and homogeneous conjugates have been developed to improve the current state of the art of the already approved and marketed ADCs. Two different approaches are especially now on the apex of the path. The first one implies the genetic modification of the antibody using the insertion of unnatural amino acids as anchor points for the conjugation [46]. The alternative viewpoint adopted the glycan-based strategy in which glycans, highly conserved in antibodies, are exploited as linking molecules for the drug, even using a click-chemistry approach [47,48].

We adopted a full-length antibody, instead of a fragment, to increase the construct size and limit the renal filtration, in future applications [49]. Elias and his colleagues highlighted the importance of optimizing the antibody density to balance effective targeting with avoiding steric hindrance and reducing site accessibility [50]. For this reason, we optimized the reaction molar ratio to obtain a final Ab/Np ratio equal to 1 and a DAR equal to 2.8 which will be improved in the modified version of this tool. According to the literature, the size of the construct (>40 nm) suggests it should take advantage of the EPR effect and be cleared through RES or hepatobiliary system after a longer circulation time concerning smaller nanoformulations [51].

In the near future, the adoption of fluorescence, due to the intrinsic feature of the employed nanoparticles, enables the finest monitoring of the fate of the construct in vivo, ex vivo, and in vitro analyses during preclinical studies on animal models, without the need for damaging radiation. The adoption of nanotools featured by different photophysical characteristics, targeting molecules, and active principles will allow for the study of complex therapeutic approaches, simplifying pharmacokinetic evaluations even in 3D models [52,53].

Ultimately, this multimodal, nanoparticle-based tool represents a possible new class of theragnostic tools. To achieve this goal, further strong work has to be conducted to fully characterize the nanotool mechanism of action and its pharmacokinetics, and especially to elucidate the cause of the loss of targeting activity at the longest incubation times.

## Data Availability

The original contributions presented in this study are included in the article/Supplementary Materials. Further inquiries can be directed to the corresponding author(s).

## Supplementary Materials

The following supporting information can be downloaded at: https://www.mdpi.com/article/10.3390/cells14090670/s1, Figure S1: Ez-flasks for monoclonal antibody production, Figure S2: Scheme of generation of a thiosuccinimide product.

## Abbreviations

The following abbreviations are used in this manuscript:

ADC	Antibody drug conjugate
ADCNP	Antibody drug conjugated nanoparticle
DAR	Drug antibody ratio
DLS	Dynamic light scattering
DMSO	Dimethyl sulfoxide
DTNB	5,5′-dithiobis(2-nitrobenzoic acid)
DTT	1,4-dithiothreitol
EDTA	Ethylenediaminetetraacetic acid
EPR	Enhanced permeability and retention
FBS	Fetal bovine serum
FTIR	Fourier transform infrared spectroscopy
IC50	Half-maximal inhibitory concentration
MES	2-(N-morpholino)ethanesulfonic acid
MMAE	Monomethyl auristatin E
MoAb	Monoclonal antibody
NEM	N-ethylmaleimide
NHS	N-hydroxysuccinimide
NP	Nanoparticle
PEG	Polyethylene glycol
PES	Polyether sulfone
PSMA	Prostate specific membrane antigen
PVDF	Polyvinylidene fluoride
SEC	Size exclusion chromatography
SMCC	Succinimidyl-4-(N-maleimidomethyl)cyclohexane-1-carboxylate
TCEP	Tris(2-carboxyethyl)phosphine
TEM	Transmission electron microscopy
TNB	5-thio-2-nitrobenzoic acid
