# Supplementary material for: Early Development of an Innovative Nanoparticle-Based Multimodal Tool for Targeted Drug Delivery: A Step-by-Step Approach
Source: Cells. 2025 May 3;14(9):670. doi: 10.3390/cells14090670 (PMC12071861; doi:10.3390/cells14090670)
Supplement: Supplementary file 1 [file cells-14-00670-s001.zip › cells-3563860-supplementary.pdf]

Early development of an innovative nanoparticle-based multimodal tool for targeted drug delivery: a step-by-step approach

Chiara Barattini[1,2], Angela Volpe[2], Daniele Gori[2], Daniele Lopez[1,3], Alfredo Ventola[2], Stefano Papa[1], Mariele Montanari[1] and Barbara Canonico[1]*

[1] Department of Biomolecular Sciences (DISB), University of Urbino Carlo Bo, 61029 Urbino, Italy
[2] AcZon srl, 40050 Monte San Pietro, Italy
[3] Department of Pure and Applied Sciences (DiSPeA), University of Urbino Carlo Bo, 61029 Urbino, Italy
* Correspondence: barbara.canonico@uniurb.it

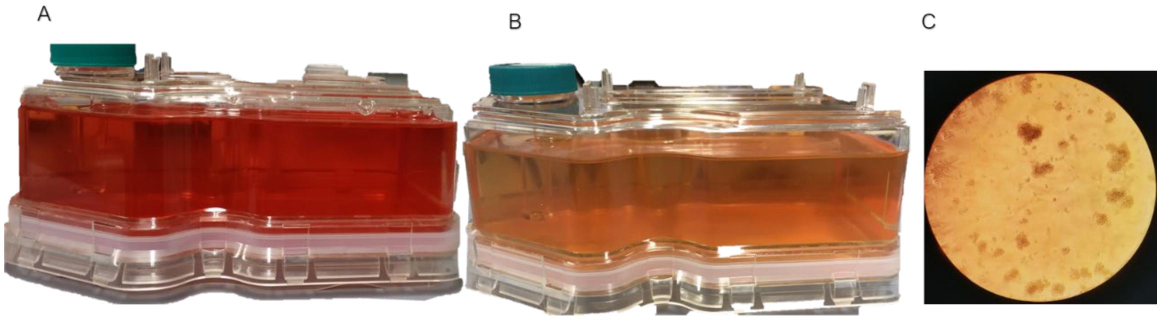

Figure S 1 – (A) Ez-flask at day 0 after the inoculum **(B)** Ez-flask at day 30 after the inoculum **(C)** Cell image after 30 days of incubation

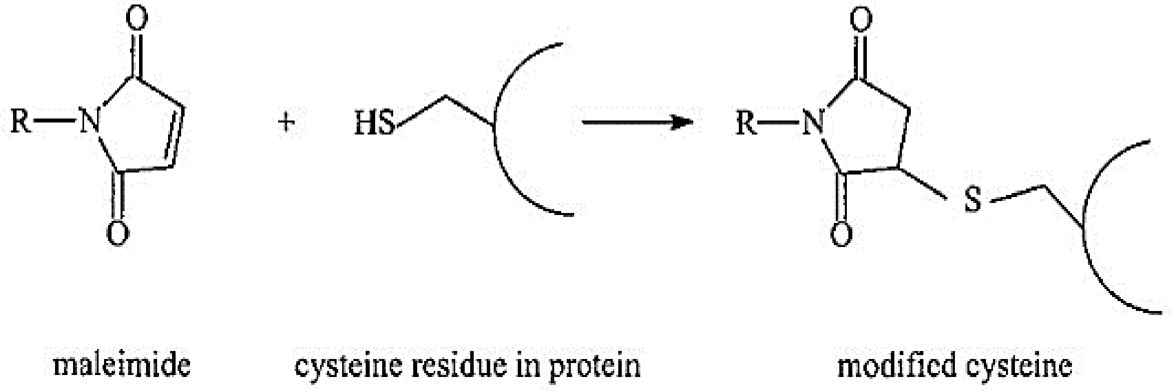

Figure S 2 - Reaction of a maleimide with a thiol

Copyright: © 2025 by the authors. Submitted for possible open access publication under the terms and conditions of the Creative Commons Attribution (CC BY) license (https://creativecommons.org/licenses/by/4.0/).
